# A novel method to establish the rabbit model of knee osteoarthritis: intra-articular injection of SDF-1 induces OA

**DOI:** 10.1186/s12891-021-04188-7

**Published:** 2021-04-03

**Authors:** Canzhang Li, Yinhong He, Yanlin Li, Guoliang Wang, Dejian Liu, Guofeng Cai, Chuan He

**Affiliations:** Departments of Sports Medicine, The First Affiliated Hospital, Kunming Medical University, Kunming, Yunnan 650032 P.R. China

**Keywords:** Osteoarthritis, Intra-articular injection, SDF-1, Animal model

## Abstract

**Background:**

Animal model of Knee Osteoarthritis (OA) is the primary testing methodology for studies on pathogenic mechanisms and therapies of human OA disease. Recent major modeling methods are divided into artificially induced and spontaneous. However, these methods have some disadvantages of slow progression, high cost and no correlation with the pathogenesis of OA.

**Methods:**

Our studies attempted to find a rapid, easy, and consistent with the natural pathological process of OA modeling method by intra-articular injection of stromal cell-derived factor 1 (SDF-1) in the rabbit knee. After induction we collected cartilage specimens from the medial femoral condyle to undergo macroscopic, histological, immunohistochemical, and biochemical evaluations. Meanwhile, compared with Hulth surgical method to evaluate its efficacy.

**Results:**

Macroscopic observation and modified Mankin score of histological staining exhibited typical features of middle stage OA cartilage in SDF-1 injected groups. Immunohistochemically, the positive expression of interleukin-1 (IL-1) and tumor necrosis factor α(TNF-α) was earlier and higher in high dose SDF-1 group than the surgical group. The matrix metalloproteinases (MMPs) in synovial fluid and chondrocytes significantly increased, but type II collagen (COLII) and aggrecan (ACAN) protein expressions decreased in SDF-1 injected group following the extension of time and increase of SDF-1 concentration.

**Conclusions:**

Our data indicated intra-articular injection of SDF-1 (40μg/kg, three times for 12 weeks) can induce rabbit knee OA model successfully more rapidly and easily than traditional surgical modeling. The study provided a further option for the establishment of knee OA animal model.

**Supplementary Information:**

The online version contains supplementary material available at 10.1186/s12891-021-04188-7.

## Background

Knee Osteoarthritis (OA), a most common joint disorder in the elderly, which is characterized include joint degeneration, loss of cartilage, and alterations of subchondral bone is the most common form of arthritis, with the highest morbidity rate of any illness. More than 40 million Americans have OA, including 80% of persons older than 50 years [1]. The prevalence rate of knee osteoarthritis in Chinese aged over 40 years old reached 15.6% [2]. The causes of OA are unknown, but it is reported that obesity, bone mass, joint injury, instability, trauma, joint deformity and age are common factors in OA, especially in the hip and knee joints [3]. Many studies have suggested that OA affects many joint structures, including degeneration of cartilage, abnormal bone remodeling and synovial inflammation, and pathobiology is far more complex [4]. To explore the pathobiology of OA, early detection and intervention are important roles to prevent and treat OA.

Animal models research is an important method to study OA pathology, including artificially induced and spontaneous models. Spontaneous model which has naturally occurring and genetically modified models that develop OA is the perfect approach similar to the OA pathological process, but it requires a long modeling period and high cost [5, 6]. Compared with spontaneous models, artificially induced modeling has a rapid progression, including biochemical or surgical induction. Surgical modeling has a high induction rate and good modeling stability, but it has a great impact on the biochemistry and high risk of joints infection due to surgical trauma and joint instability. Hulth surgical modeling is the most classic and popular method [7]. Biochemical modeling method, intra-articular injection chemicals such as papain and collagenase, shows high results in relatively homogeneous degree and reproducibility of a pathological state in a short period. However, the method exists controversy about the pathophysiology, which biochemical model might induce significant amount of inflammation and also may not represent naturally occurring OA state found in human [8].

Stromal cell derived factor-1 (SDF-1), produced by synovial cells in OA joint, is a cytokine associated with inflammation. Once SDF-1 binds to its ligand C-X-C chemokine receptor type 4 (CXCR4), a G protein-coupled receptor located in the surface of chondrocytes, it will activate multiple downstream signaling pathways to induce chondrocyte apoptosis and release MMPs to degrade extracellular matrix (ECM) causing cartilage degradation and increasing the pathological process of OA [9–11]. Some studies showed that the SDF-1/CXCR4 signaling pathway plays a critical role in the pathogenesis of OA cartilage degeneration and a remarkable increase of SDF-1 in OA patients` synovial fluid [11, 12]. Xu et al. demonstrated that the SDF-1 level was associated with the radiographic severity of OA [13]. Our previous studies found CXCR4 inhibitors AMD3100 and TN14003 could block the SDF-1/CXCR4 axis and reverse the OA process [12, 14].

Therefore, it was assumed that exogenous SDF-1 could be used to simulate the pathological process of OA, and establish OA animal model. Our experiment tries to use SDF-1 intra-articular injection to induce OA animal models, explore the appropriate dose and time for modeling and compare with Hulth surgical modeling in morphology, histology and molecular biology in order to find a modeling method more similar to the natural OA pathological process.

## Results

### Gross observation

**Fig. 1:**
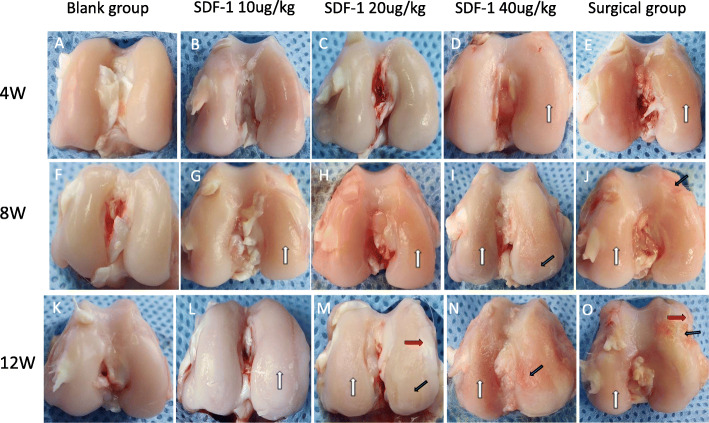
Macroscopic observation of cartilage specimens 4,8,12 weeks later after intra-articular injection of SDF-1 and surgery. **a**, **f**, **k** saline injection knees; **b**-**d**, **g**-**i**, **l**-**n** 20μg/kg to 40μg/kg SDF-1 injection knees; **e**, **j**, **o** surgical knees. White arrows: Dry and roughness of the cartilage surface. Black arrows: Cartilage defection. Red arrows: Osteophytes formed at the joint margins

After 4 weeks of injection, there was no significant difference in cartilage surfaces morphology between the experimental groups and the blank group. (Fig. 1a-e). After 8 weeks, the cartilage surface of medial femoral condyles in the experimental groups showed roughness and dry, the gloss dulling, and even some cartilage defections appeared in SDF-1 40μg/kg group and the surgical group compare with the blank group (Fig. 1f–j). Joint deformation and cartilage defection were extreme and osteophytes formed at the joint margins in 20μg/kg, 40μg/kg SDF-1 group and the surgical group after 12 weeks (Fig. 1k–o). The severity of cartilage degeneration demonstrates in a dosage dependent manner in various concentrations of SDF-1 injected groups, and SDF-1 40μg/kg group was similar to the surgical group.

### Histological evaluation and modified Mankin score

**Fig. 2:**
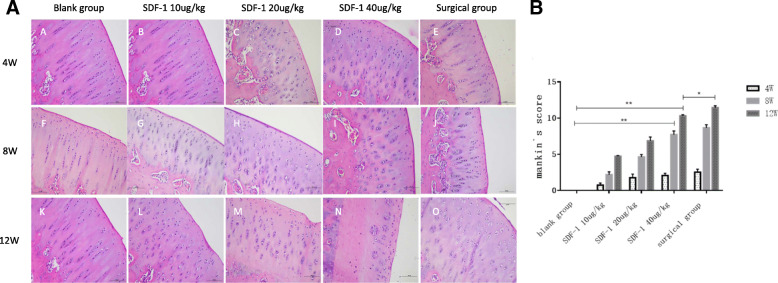
**A** HE staining of osteochondral specimens. A, F, K: Normal saline injected knees; B-D, G-I, L-N: 20μg/kg to 40μg/kg SDF-1 injected knees; E, J, O: surgical knees. Scale bars = 500 μm. **B** The modified Mankin score. Data were measured as mean ± standard error of the mean. (*p < 0.05, **p < 0.01 and ***p < 0.001)

After 4 weeks, Hematoxylin-eosin (HE) staining of osteochondral specimens from the intermediate region of the medial femoral condyle in experimental groups demonstrated slightly cells swelling, the polarity disappearing, the structural disorganization and the number of chondrocytes significantly decreased with a ragged edge (Fig. 2A B-E) compared with blank group (Fig. 2A A). After 8 weeks, the above phenomenon was even more obvious, cartilage surface thinness and deep fissures were observed (Fig. 2A F-J). After 12 weeks, chondrocytes of experimental groups were partly lost or arranged in clusters, cartilage defects were detected, and hypertrophic chondrocytes and deep cracks were visible (Fig. 2A K-O). Chondrocytes were completely missing from the middle and deep layers of cartilage in SDF-1 40μg/kg group (Fig. 2A N). The modified Mankin’s score was significantly higher in SDF-1 groups than in the blank group after 8 and 12 weeks. At 12 weeks, modified Mankin score of SDF-1 40μg/kg group reached the middle stage of OA [15] which is beyond the surgical group (*P* < 0.05) (Fig. 2B).

### Immunohistochemical evaluation

**Fig. 3:**
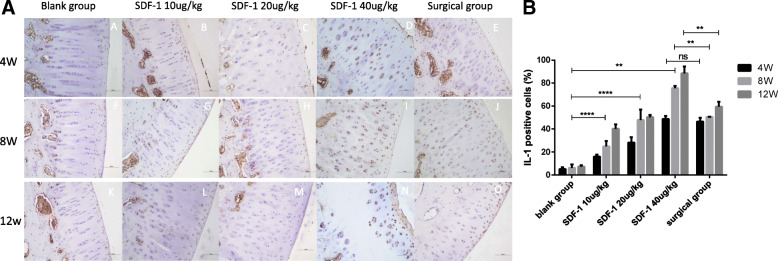
**A** IL-1 staining of osteochondral specimens. A, F, K: Normal saline injected knees; B-D,G-I,L-N: 20μg/kg to 40μg/kg SDF-1 injected knees; E, J, O: surgical knees. Scale bars = 500 μm. **B** The percentages of IL-1 positive cells were randomly counted using three fields. Data were measured as mean ± standard error of the mean. (*p < 0.05, **p < 0.01, ***p < 0.001, ****p < 0.0001, and ns: no significant difference)

**Fig. 4:**
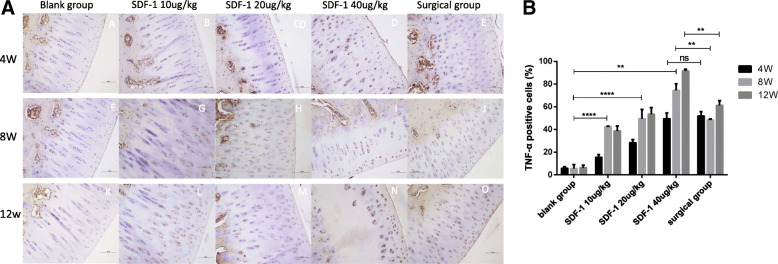
**A** TNF-α staining of osteochondral specimens. A, F, K: Normal saline injected knees; B-D, G-I, L-N: 20μg/kg to 40μg/kg SDF-1 injected knees; E, J, O: surgical knees. Scale bars = 500 μm. **B** The percentages of TNF-α positive cells were randomly counted using three fields. Data were measured as mean ± standard error of the mean. (*p < 0.05, **p < 0.01, ***p < 0.001, ****p < 0.0001, and ns: no significant difference)

IL-1 and TNF-α immunohistochemical staining were performed to evaluate secretion levels of inflammatory factors in cartilage tissue. It’s not obvious at 4 weeks (Figs. 3A A-E, 4A A-E), but the positive expression of IL-1 and TNF-α in the experimental groups was significantly increased in SDF-1 injected and surgical cartilage after 8 weeks. Chondrocytes presented that the polarity disappeared, the structure disorganized and arranged in clusters (Figs. 3A F-J, 4A F-J). At 12 weeks Extracellular matrix (ECM) staining was uneven, resulting in cracks and severe damage (Figs. 3A K-O, 4A K-O). At 4 weeks, the positive expression of IL-1 and TNF-α in the surgical group was higher than SDF-1 10μg/kg and 20μg/kg groups, but close to SDF-1 40μg/kg group (ns). After 8 weeks it was lower than SDF-1 40μg/kg group (*P* < 0.01) (Fig. 3B, 4B).

### The content of MMP-3, 9, 13 in synovial fluid by Elisa

**Fig. 5:**
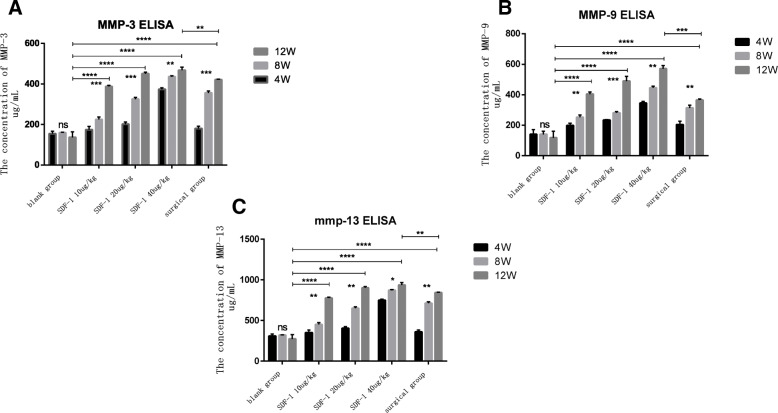
ELISA assay showed the MMPs content in the synovial fluid. **a** MMP-3. **b** MMP-9. **c** MMP-13. Values are presented as mean ± SD. (*p < 0.05, **p < 0.01, ***p < 0.001, ****p < 0.0001, and ns: no significant difference)

ELISA revealed SDF-1 injection significantly elevated the content of MMP-3, 9, 13 in the synovial fluid which is catabolic proteases associated with OA and the main disruptive factors in cartilage degeneration. MMP-3, 9, 13 increased with the extension of time in different concentration SDF-1 injected groups and the surgical group (*p* < 0.01). At the same time point, MMP-3, 9, 13 of SDF-1 injection groups went up with increase of drug administration concentration (*p* < 0.01). Compared with the surgical group, the content of MMP-3, 9, 13 in SDF-1 20μg/kg group and SDF-1 40μg/kg group was higher than surgical group at the same time point (*P* < 0.01). However, the growth of MMP-13 in the synovial fluid became slow gradually when up to a certain level after 8 weeks (Fig. 5a, b, c).

### MMP-3,9,13 mRNA expressions in cartilage of OA model rabbit

**Fig. 6:**
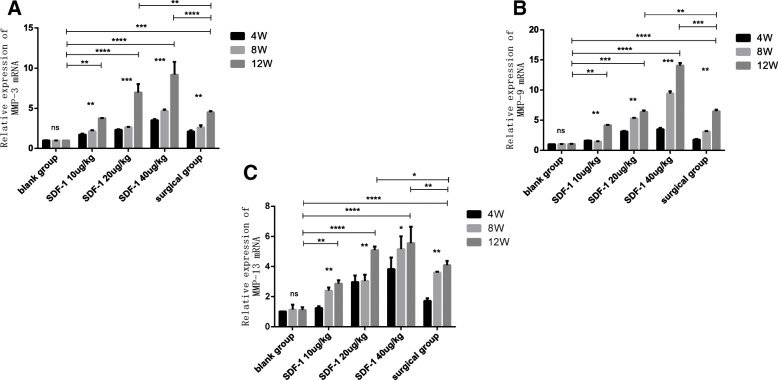
The expression of MMPs mRNA in cartilage at 4, 8 and 12 weeks after treatment by qPCR. **a** MMP-3. **b** MMP-9. **C:** MMP-13. Values are the means ± SD. (*p < 0.05, **p < 0.01, ***p < 0.001, ****p < 0.0001, and ns: no significant difference)

The expressions of MMP-3, 9, 13 mRNA in articular cartilage after 4,8 and 12 weeks after treatment were determined by qPCR. The MMP-3, 9, 13 mRNA expressions in the surgical group and the SDF-1 injected groups were all increased with the extension of time compared with the blank group (*p* < 0.01). After 12 weeks, MMP-3, 9, 13 expressions in the SDF-1 20μg/kg and 40μg/kg groups were superior to the surgical group (*p* < 0.01). After 8 weeks, MMP-13 mRNA expression in SDF-1 40μg/kg group increase became slow as same as Elisa test (Fig. 6a, b, c).

### The effect of SDF-1 on COL II、ACAN in cartilage

To evaluate the effect of SDF-1 induced OA model, we investigated the expression of COLII、ACAN in cartilage by qPCR and western blot analysis.

**Fig. 7:**
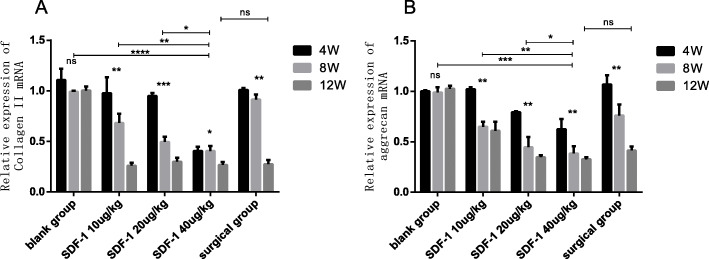
COLIImRNA and ACAN mRNA expression in cartilage at week 4,8 and 12 after treatment. **a** COLIImRNA. **b** ACAN mRNA. Values are the means ± SD. (*p < 0.05, **p < 0.01, ***p < 0.001, ****p < 0.0001, and ns: no significant difference)

QPCR demonstrated the expression of COLIIand ACAN mRNA in the blank group did not change significantly with the extension of time, but decreased in the experimental groups (*p* < 0.01). At the same time point, the expression of COLIIand ACAN mRNA in the different concentration SDF-1 injected groups reduced with the rise of SDF-1 concentration (*p* < 0.01). After 12 weeks, the mRNA expression in SDF-1 40μg/kg groups had no significant difference compared with the surgical group (ns) (Fig. 7a, b).

**Fig. 8:**
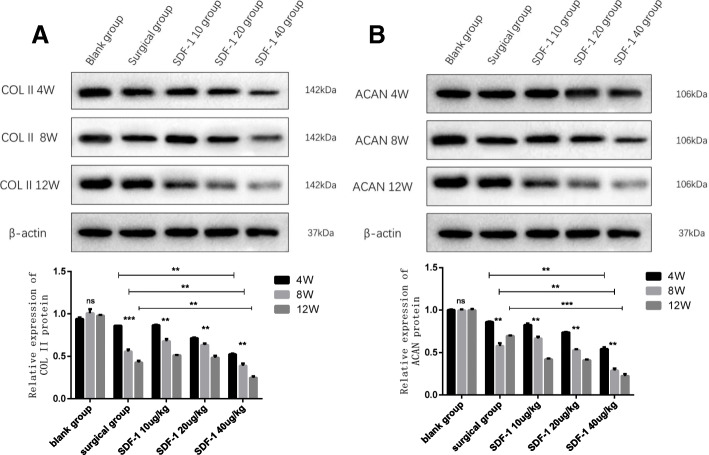
COLIIand ACAN protein expressions were assessed in cartilage using western blotting. **a** COLIIprotein. **b** ACAN protein. The image was edited with Photoshop CS6. Full-length blots/gels are presented in Supplementary Figure [8A-B original]. Data are presented as the mean ± standard error of the mean. (*p < 0.05, **p < 0.01, ***p < 0.001, ****p < 0.0001, and ns: no significant difference)

Western blot analysis showed COLIIand ACAN protein expressions diminished over time in experimental groups (*p* < 0.01). COLIIand ACAN protein expressions reduced on a dose-dependent manner in different concentration SDF-1 injected groups (*p* < 0.01). Moreover, COL II and ACAN protein expressions in SDF-1 40μg/kg group are lower than the surgical group at the same time point in time (*p* < 0.01) (Fig. 8a, b).

## Discussion

Although, extensive animal models for OA study have been developed over the past half century, there is no gold standard animal model used in OA research. In the ideal animal model, the disease must be induced reliably, with 100% penetrance, and within a suitable time frame, and yet still present with disease characteristics that are comparable to the human condition [16, 17]. In this study we found that intra-articular injection of SDF-1 as a novel biochemical method can successfully induce knee OA in rabbits, and the effect of SDF-1 40μg/kg injection after 12 weeks is similar to the classic surgical modeling. As an important chemokine produced naturally in the pathological process of human OA, using SDF-1 for modeling is more consistent with the pathology of OA than other biochemical modeling.

Macroscopic observation and HE staining of SDF-1 treated group in our experiment showed typical characteristics of early and middle OA [18]. Even chondrocytes in SDF-1 40μg/kg group were completely missing from the middle and deep layers of cartilage. It indicated SDF-1 in high concentrations may affect the proliferation of chondrocytes. Mark C. Lee’s previous study got a similar result in when administration of SDF-1 in rabbit cartilage growth plate disrupted the columns of chondrocytes and closed the growth plate growth [19]. Modified Mankin score showed a histological deterioration of cartilage by SDF-1 injection and the degeneration of articular cartilage went to middle stage of OA in SDF-1 40μg/kg group after 12 weeks which are more serious than the surgical group.

As pivotal pro-inflammatory cytokines in AO pathology, IL-1 and TNF-α are important in promoting cartilage matrix degradation [20, 21]. In the immunohistochemical study, the positive expression chondrocytes of IL-1 and TNF-α are significantly increased in all SDF-1 treated groups. After 8 weeks, the positive expression chondrocytes of IL-1 and TNF-α in SDF-1 40μg/kg group were more than the surgical group. It indicated high dose SDF-1 intra-articular injection promotes inflammatory factors secretion earlier than Hulth surgery modeling, and inflammation was more severe. Wang et al. also demonstrated that activation of SDF-1/CXCR4 pathway significantly promoted the secretion of IL-1 β, IL-6, IL-15, IL-17, IL-18, and TNF-α. These inflammatory factors activate MAPKs pathway and TLRs pathway to promote the apoptosis of chondrocytes, and finally lead to the further imbalance of homeostasis in the knee joint [22].

MMPs play a key role in the degradation of cartilage matrix and destructing the stability of articular cartilage, especially MMP-3,9,13 [23–25]. MMPs are rarely expressed under normal physiological conditions, however significantly overexpressed in synovial fluid and articular cartilage of human OA [26]. We examined the secretion level of MMP-3,9,13 in synovial fluid and the expressions of MMPs mRNA in cartilage. Either in joint fluid or cartilage, MMP-3,9,13 raised with increase of SDF-1 injection concentration and time. At 12 weeks, the secretion level and mRNA expression of MMP-3,9,13 in SDF-1 40μg/kg group were even beyond the surgical group. After 8 weeks, the growth of MMP-13 in the SDF-1 40μg/kg group became slow gradually demonstrated that the modeling experiment only went to the middle stage of OA. As previous research reported, MMP-13 is abnormally expressed in different stages of the OA process which is upregulated in the early stage of OA cartilage and downregulated in the late stage [27]. Similarly, Wang, G et research showed TN14003 can target blocking SDF-1/CXCR4 signaling pathway in vivo, reduce the expression and secretion of MMP-3, MMP-9, and MMP-13 in cartilage tissue, and reduce the degradation of collagen II and aggregating proteoglycan, thus delaying the degeneration of articular cartilage [28].

Col II and ACAN are the principal component of cartilage ECM, and their content is the best indicators of cartilage degeneration [29]. In our study, the expression of Col II and ACAN mRNA and protein was downregulated with the increase of SDF-1 injected concentration and time. The reduction of Col II and ACAN in SDF-1 40μg/kg injected group after 8 weeks were more than the surgical group. This result revealed high dose SDF-1 injection leads to ECM degradation more rapidly and seriously which means the time of building OA animal model with SDF-1 is shorter compared with classical surgical method. Other researchers also obtained similar results as our experiment. By changing the content of SDF-1 in the joint, SDF-1/CXCR4 signaling pathway can be regulated, which affects the apoptosis of chondrocyte, and inflammation, and matrix catabolism [29–31].

Because SDF-1 is a small molecular weight peptide (8-kDa) and its short half life, 40 μg/kg SDF-1 injection multiple times a week for 12 weeks may maintain high active concentration in the joint. It was mentioned in another paper that an infusion pump system was used for continuous release of SDF-1 in the joint [20]. However, whether high concentration of SDF-1 has any effect on rabbits, we had made close observation. In the course of the experiment, all rabbits were alive, no adverse reactions and complications of joint such as infection and instability occurred in surgical modeling [32, 33].

The results of current research were all highly consistent with the predictions that exogenous SDF-1 intra-articular injection could establish the OA rabbit model. High concentration of SDF-1 (40μg/kg) can cause the degeneration of rabbit knee joints more quickly reaching to middle stage of OA and the efficacy of SDF-1 injection is better than traditional surgical modeling. It provides a new method for modeling animals knee joint.

Some limitations remain in the present study. Firstly, only 12 weeks were observed in the current study, and the pathological features at the early and middle stages of OA were mainly presented in our model. Whether the model is applicable to the later stage of OA still needs a longer period of observation. Secondly, OA is a complex disease and the pathological changes of OA are the result of many cytokines and chemokines synergistic effect. Only SDF-1 was used to induce modeling, which may ignore the influence of other factors. Thirdly, this experiment has not been compared with other chemical modeling methods, and cannot fully explain the advantages of the same type of modeling methods. Further studies of SDF-1 induced OA model will be carried out to overcome above shortages in the future.

## Conclusion

The present study demonstrated that intra-articular injection of SDF-1 can induce the release of inflammatory cytokines, MMPs secretion increased, the inhibition of ACAN, COL II synthesis, and accelerated extracellular matrix degradation. SDF-1 40μg/kg injection three times for 12 weeks may be an appropriate approach to induce OA rabbit knee model more rapidly and easily than traditional surgical modeling.

## Materials and methods

### Animals and reagents

All male 3-month-old Japanese white rabbits (weighting 2–2.5 kg) were obtained from the Experimental Animal Department of Kunming Medical University (Kunming, China) and were adapted for 1 week prior to the experimental procedures. The Institutional Ethics Committee of Kunming Medical University approved all experimental protocols, using an animal model. All surgical procedures were performed in accordance with Guidelines for the ethical review of laboratory animal welfare People’s Republic of China National Standard GB/T 35892–2018 (Issued 6 February 2018 Effective from 1 September 2018). This study was carried out in compliance with the ARRIVE guidelines.

The SDF − 1 alpha human species,1000μg/ml, which was purchased from Pepro Tech Co., Ltd. (Rocky Hill, America) used in the present experiment was diluted to different concentrations mixing with normal saline. The 3,3′-diaminobenzidine (DAB) Substrate kit was purchased from ZSGB-Bio Co., Ltd. (Beijing, China), and Hematoxylin-Eosin/HE Staining Kit was purchased from Boster Biological Technology Co. Ltd. (Wuhan, China).

### Rabbit OA model and SDF-1 treatment

Forty-five rabbits were randomly allocated into five groups: Normal saline (blank group), SDF-1 low dose (10μg/kg group), SDF-1 medium dose (20μg/kg group) and SDF-1 high dose (40μg/kg group), surgery (surgical group). The knees of the blank group and SDF-1 groups rabbits’ right hind legs were injected with 0.2 ml volumes of saline or various concentrations of SDF-1, three times a week interval of 1 day. The surgical group was performed with classical surgical modeling method, Hulth method, which cuts off medial collateral ligament, anterior cruciate ligament, and posterior cruciate ligament of the knee joint and removes medial meniscus. Penicillin (20,000 U/kg) was injected daily for 3 days after the operation to prevent infection [34]. All experimental rabbits were fed normally and motivated limbs movement. Throughout the modeling process, the behavior and weight changes of rabbits were observed. Four, eight, twelve weeks after operation, three rabbits from each group were sacrificed under intravenous anesthesia, and the sample was collected from experimental knees.

### Macroscopic observation and histological assessment

After sacrifice, right knee joints were dissected and the gross appearance of the femoral condyle was assessed and photographed. Osteochondral samples were collected from the intermediate region of the medial femoral condyles and fixed with 4% neutral formalin for 48 h, decalcified with 14% EDTA (Hyclone Co., Ltd.,Logan, Utah, America) for 15 days, dehydrated through a series of graded ethanol solutions, cleared in xylene, and embedded in paraffin. Sections, 5 μm in thickness, were stained with hematoxylin-eosin (H&E) to examine tissue morphology. Histological samples were graded using a modified Mankin score in Table 1.

**Table 1:** modified Mankin score (criteria for histological evaluation)

Item	Grade/classification	Grade
Structural intergrity	Normal	0
	Irregular Surface	1
	Pannus formation	2
	Fissures into transitional layer	3
	Fissures into emmiting layer	4
	Fissures into calcified layer	5
	Complete disorganization	6
	Cells Normal	0
	Hypercellularity	1
	Cloning	2
	Hypocellularity	3
Hematoxylin-eosin staining	Normal	0
	Slight reduction	1
	Moderate reduction	2
	Severe reduction	3
	No dye noted	4
Tidemark intergrity	Normal	0
	Disruption	1

### Immunohistochemical evaluation

After rehydrating with PBS, deparaffinized sections were quenched endogenous peroxidase activity with H2O2 for immunological staining. Then sections were blocked with 5% normal goat serum (Bioss, Beijing, China) in PBS and incubated with primary antibodies against IL-1 (Bioss, Beijing, China) and TNF-α (Bioss, Beijing, China) overnight at 4 °C. The osteochondral were incubated with the second antibody and biotin-labeled horseradish peroxidase after incubation overnight at 4 °C with the primary antibody (1:200). Subsequently, the antibody binding was visualized with a 3, 3′-diaminobenzidine tetrahydrochloride (DAB) kit (Boster, China) before brief counterstaining with hematoxylin. Finally, staining results were evaluated using light microscopy (Nikon, Japanese).

### Elisa

Before dissecting knee joint, the synovial fluid was extracted with a 1 mL syringe and put into a 1 mL centrifuge tube frozen and stored. The content of MMP-3, 9, 13 in synovial fluid was measured by ELISA according to the ELISA kit (R&D Systems, Minnesota, USA) manufacturer’s instructions. Absorbance at a wavelength of 450 nm was detected using an UV-2600 microplate reader (Shimadzu Corporation, Kyoto, Japan).

### Real-time quantitative reverse transcription-polymerase chain reaction (qRT-PCR) analysis

To evaluate the effect of SDF-1 on OA chondrocytes, the expressions of arthritis-related genes. MMP-3, 9, 13, aggrecan, type II collagen mRNA expression of OA cartilage were detected by qRT-PCR. For the in vivo study, cartilages collected at 4, 8 and 12 weeks were pulverized in liquid nitrogen. TRIZOL reagent (Lifetech Limited, USA) was added to lyse the cells. Chondrocytes were washed once with PBS and the total RNA was extracted using a RNA isolation kit (Lifetech Limited, USA) according to the manufacturer’s instructions. Using a reverse transcription kit (Fermentas Company, USA), approximately 2 μg total RNA was used as a template and reverse transcribed into cDNA. The qRT-PCR reactions were carried out using a Quantitative PCR Detection System (Realplex 4, Eppendorf Corporation, USA) with FastStart Universal SYBR Green Master (Kapa Biosystems, Boston, USA). PCR thermocycling conditions were as follows: 10 min at 95 °C, 15 s at 95 °C and 30 s at 60 °C. The primers used for PCR are shown in Table 2. RNA expression levels were calculated by using the 2^-ΔΔCt^ method and glyceraldehyde-3-phosphate dehydrogenase (GAPDH) served as the internal reference.

**Table 2:** Sequences of polymerase chain reaction primers used for the detection of miRNA expression

Name	Sequence	Length
GAPDH-F	TTAACTCTGGCAAAGTGGAT	89
GAPDH-R	GGTGGAATCATACTGGAACA	
MMP3-F	CCAGACATATAGCAGAAGAC	106
MMP3-R	AACTCCGACTGTGAAGAT	
MMP9-F	TCTGCAACGTGAATGTCT	75
MMP9-R	AGTACCTCCCATCCTTGA	
MMP13-F	AACAGTAACGAGGATGAT	81
MMP13-R	CAGAGGATGGTAGTATGAT	
Collagen II-F	AGAGGTATAATGATAAGGAT	100
Collagen II-R	GTGTCTTCACAGATTATG	
Aggrecan-F	GTAGTGGTGAATCTTCTG	86
Aggrecan-R	TAAGCCTTCTTCTTCTCT	

### Western blot analysis

Protein expressions of type II collagen and aggrecan were measured by western blot. To prepare total protein from cartilage tissues obtained at 4, 8 or 12 weeks after treatment. Before aggrecan protein quantification, each sample was deglycosylated with chondroitinase ABC (ChABC) in PBS with acetic acid (50 U/ml, pH 7.8) for 24 h at 37 °C to remove GAG side chains from the aggrecan core protein. The bicinchoninic acid assay method was used to assess the protein quantity and quality. Protein samples (80 μg) were separated by SDS-polyacrylamide gel (3 h) and transferred to polyvinylidene difluoride membrane (wet method, 200 mA current for 60 min). PVDF membranes were blocked in 5% skimmed milk powder for 1 h, followed by incubating with primary antibodies (Abcam, Cambridge, UK; anti-Col II at 1:1000, anti-aggrecan at 1:1000 or anti-β-actin at 1:5000) overnight at 4 °C. After washing three times with TBST, the membranes were incubated with secondary antibodies (Abcam, Cambridge, UK; anti-mouse or anti-rabbit; dilution, 1:8000) for 2 h at room temperature. Subsequent to TBST washing three more times, the ECL reagent (Thermo Fisher Scientifc, Inc.) was added for 1–3 min incubation in the dark. The band intensities were quantified by densitometry with ImageJ 1.46r software (National Institutes of Health, Bethesda, MD, USA).

### Statistical analysis

All the data are presented as the mean ± standard deviation. Differences between two groups were analyzed by unpaired t-test or the Mann-Whitney U test for experiments in which the datasets were not normally distributed. Differences in multiple groups were analyzed by ANOVA. GraphPad Prism 6 software (GraphPad Software, La Jolla, CA, USA) was used for all statistical analyses. *P* values < 0.05 were considered statistically significant. All experiments were repeated at least three times.

## Supplementary Information

**Additional file 1.**

## Data Availability

The datasets used and/or analysed during the current study are available from the corresponding author on reasonable request.

## Abbreviations

OA: Osteoarthritis
SDF-1: Stromal cell-derived factor 1
CXCR4: C-X-C chemokine receptor type 4
MMPs: Matrix metalloproteinases
HE: Hematoxylin-eosin
DAB: 3,3′-diaminobenzidine tetrahydrochloride
COLII: Type II collagen
ACAN: Aggrecan
