# Supplementary figures and images for: A novel method to establish the rabbit model of knee osteoarthritis: intra-articular injection of SDF-1 induces OA
Source: BMC Musculoskelet Disord. 2021 Apr 3;22:329. doi: 10.1186/s12891-021-04188-7 (PMC8019508; doi:10.1186/s12891-021-04188-7)

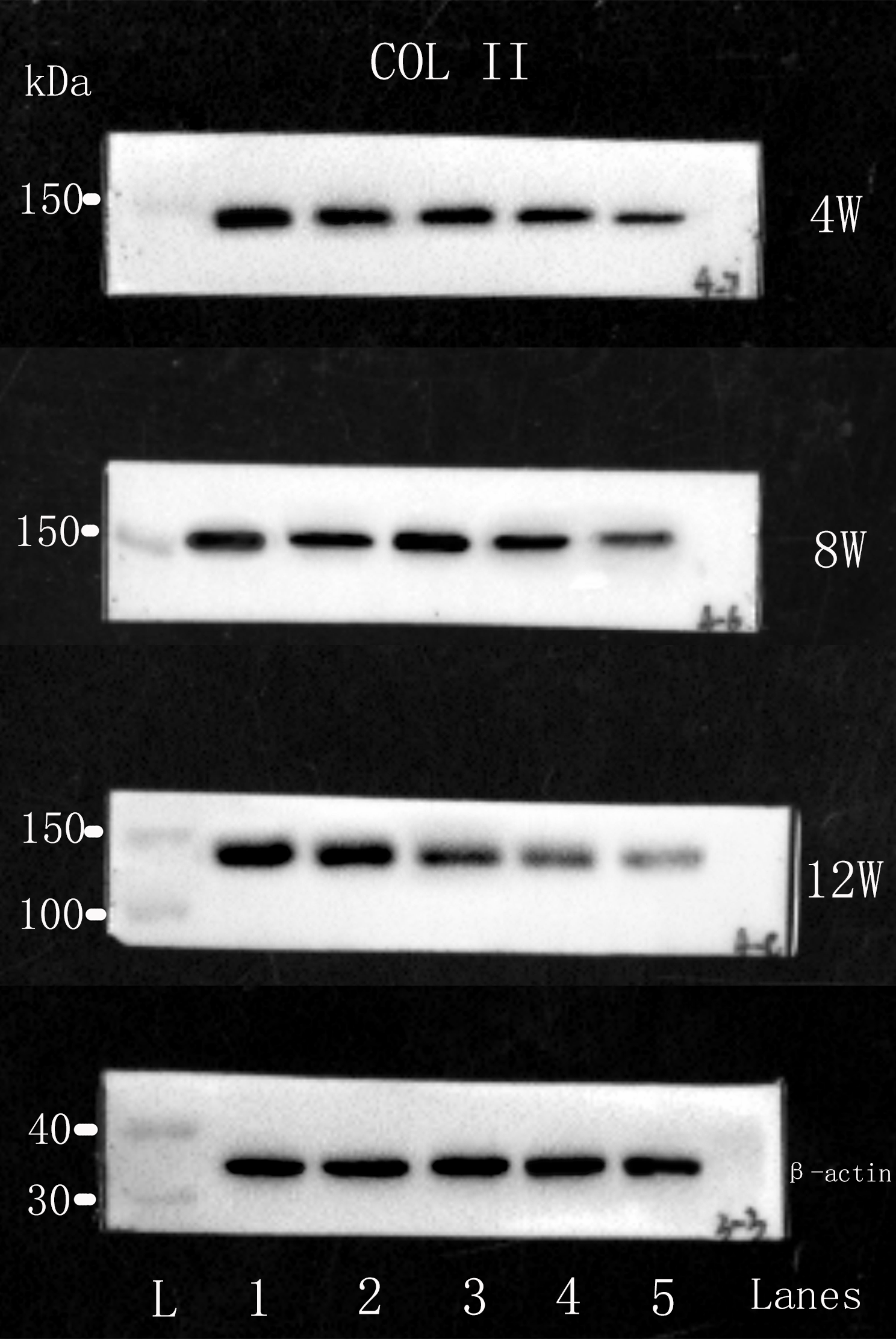

Supplement: Supplementary file 1 — Additional file 1. [file 12891_2021_4188_MOESM1_ESM.zip › Figure 8A B Original/Figure 8A original.tif]

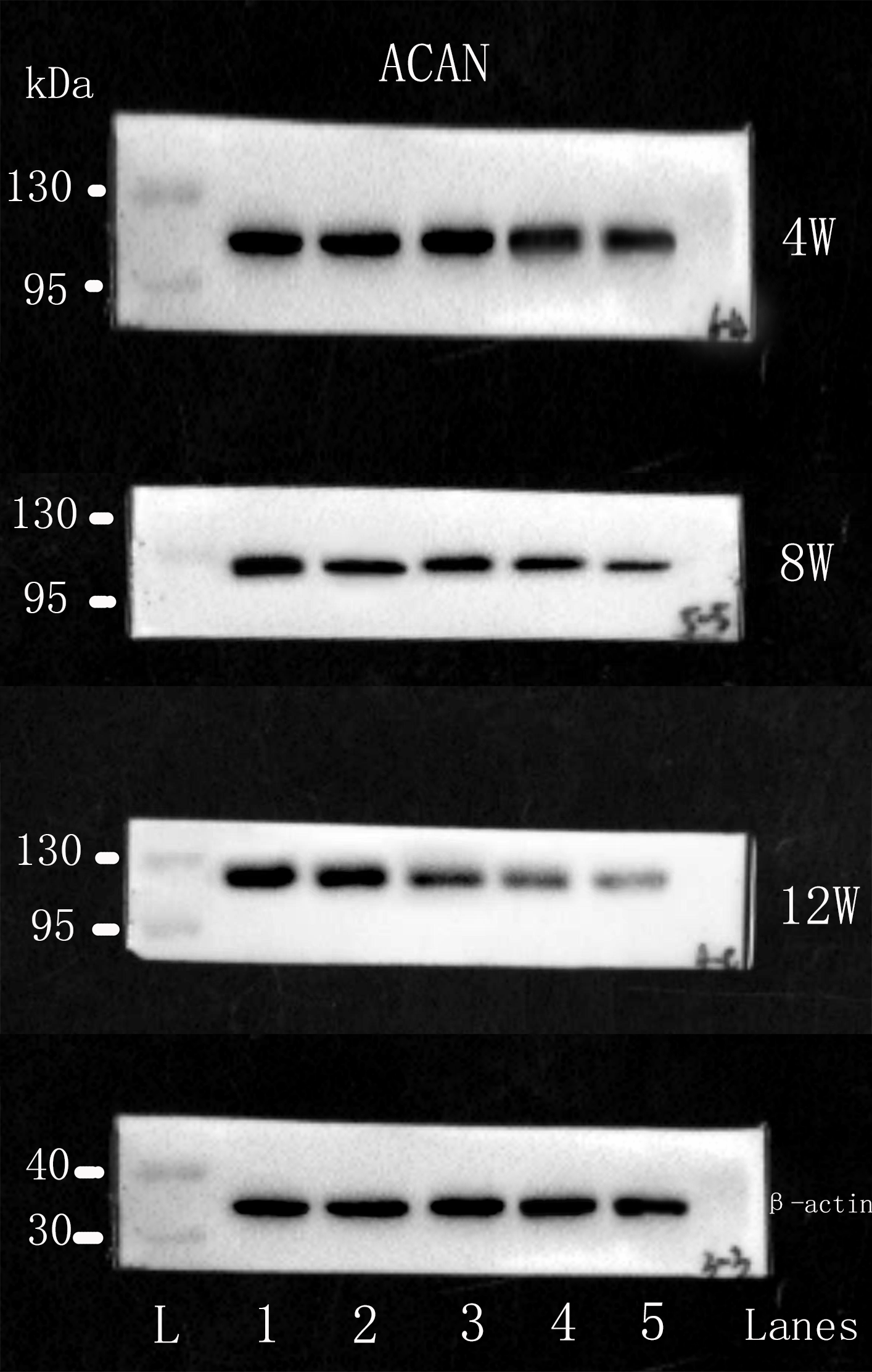

Supplement: Supplementary file 1 — Additional file 1. [file 12891_2021_4188_MOESM1_ESM.zip › Figure 8A B Original/Figure 8B original.tif]
